# m^5^C Regulator-mediated methylation modification clusters contribute to the immune microenvironment regulation of multiple myeloma

**DOI:** 10.3389/fgene.2022.920164

**Published:** 2022-08-25

**Authors:** Hefei Ren, Chang Liu, Hongkun Wu, Zhenhua Wang, Sai Chen, Xiaomin Zhang, Jigang Ren, Huiying Qiu, Lin Zhou

**Affiliations:** ^1^ Department of Laboratory Medicine, Shanghai Changzheng Hospital, Naval Medical University, Shanghai, China; ^2^ Department of Hematology, Shanghai General Hospital, Shanghai Jiao Tong University School of Medicine, Shanghai, China

**Keywords:** multiple myeloma, 5-methylcytosine, immune microenvironment, immune therapy, biological function

## Abstract

**Background:** Multiple myeloma (MM) is a hematological malignancy in which plasma cells proliferate abnormally. 5-methylcytosine (m^5^C) methylation modification is the primary epigenetic modification and is involved in regulating the occurrence, development, invasion, and metastasis of various tumors; however, its immunological functions have not been systematically described in MM. Thus, this study aimed to clarify the significance of m^5^C modifications and how the immune microenvironment is linked to m^5^C methylation in MM.

**Method:** A total of 483 samples (60 healthy samples, 423 MM samples) from the Gene Expression Omnibus dataset were acquired to assess the expression of m^5^C regulators. A nomogram model was established to predict the occurrence of MM. We investigated the impact of m^5^C modification on immune microenvironment characteristics, such as the infiltration of immunocytes and immune response reactions. We then systematically evaluated three different m^5^C expression patterns to assess immune characteristics and metabolic functional pathways and established m^5^C-related differentially expressed genes (DEGs). In addition, biological process analysis was performed and an m^5^C score was constructed to identify potentially significant immunological functions in MM.

**Result:** Differential expressions of m^5^C regulators were identified between healthy and MM samples. The nomogram revealed that m^5^C regulators could predict higher disease occurrence of MM. We identified three distinct m^5^C clusters with unique immunological and metabolic characteristics. Among the three different m^5^C clusters, cluster C had more immune characteristics and more metabolism-related pathways than clusters A and B. We analyzed 256 m^5^C-related DEGs and classified the samples into three different m^5^C gene clusters. Based on the m^5^C and m^5^C gene clusters, we calculated m^5^C scores and classified each patient into high- and low-m^5^C score groups.

**Conclusion:** Our study demonstrated that m^5^C modification is involved in and contributes to the diversity and complexity of the immune microenvironment, which offers promise for the development of accurate therapeutic strategies.

## 1 Introduction

Multiple myeloma (MM) is a malignant proliferative hematological disease that originates from the hematopoietic system and is caused by the abnormal proliferation of plasma cells (Cowan et al., 2022). The disease can affect any organ of the human body, with the most frequently involved organs being the bone and bone marrow. MM accounts for 10% of the total number of patients with hematological tumors and is the second most common type of hematological malignancy globally (Allegra et al., 2022). Monoclonal immunoglobulins appear in the blood and urine of MM patients, which results in functional damage to related target organs, such as bone marrow hematopoiesis, kidneys, and bone. MM manifests clinically with anemia, bone pain and osteolytic destruction, renal failure, and repeated infections (Wang et al., 2021). Because MM tumor cells are derived from immune cells, they can affect the humoral and cellular immunity of MM patients (Jadoon and Siddiqui, 2021). In the early monoclonal gammopathy of undetermined significance (MGUS) stage of the disease, an average of 1.0–1.5% of MGUS patients per year progress to active myeloma, which indicates that the interaction between the immune system and tumor cells determines the final evolution of MM (Nakamura et al., 2020). Abnormal plasma cells in MGUS can affect various biological processes in the bone marrow microenvironment, such as cell homing, cytokine secretion, immune cell function, and angiogenesis. The homeostasis of the tumor immune microenvironment can also further promote the malignant proliferation of plasma cells. Therefore, a detailed understanding concerning the biological characteristics and immunoregulatory mechanisms of MM cells will be crucial for the discovery of new diagnostic biomarkers and possible drug targets as well as the exploration of new immunotherapy methods for MM.

As a groundbreaking and emerging area of epigenetic research, 5-methylcytosine (m^5^C) epigenetic modification has received increased attention in recent years. m^5^C methylation modification is a methylation of the fifth carbon atom on the DNA cytosine (C), in the context of cytosine-guanine dinucleotides (CpG) through the action of the DNMTs (Bestor, 1988). m^5^C modification occurs frequently in cells and plays an essential role in maintaining gene expression and genomic stability in cells. m^5^C is modified primarily by methyltransferases (“writers”), demethylases (“erasers”), and binding proteins (“readers”). A range of biological functions are exerted on determining cell differentiation state, selecting cell identity, mitochondrial activity, and immunoregulatory mechanisms. m^5^C methylation modification is a dynamic and reversible process; m^5^C “writers” catalyze the formation of m^5^C, “readers” function by recognizing and binding to m^5^C methylation sites, and “erasers” catalyze the demethylation of m^5^C (Hu et al., 2021). Several studies have suggested that m^5^C regulation contributes to underlying immune-regulating mechanisms (Wang et al., 2017; Guo et al., 2020). The abnormal expression of m^5^C regulators may represent a good candidate for modifying m^5^C in systemic lupus erythematosus CD4^+^ T cells. Moreover, in rat T lymphocytes, m^5^C writers promote the translation of interleukin 17A by methylating the C466 site.

Despite the growing evidence that m^5^C regulation plays a regulatory role in immune responses, no studies have been conducted to demonstrate the role m^5^C regulation plays in the pathogenesis of MM. Such in-depth studies of m^5^C regulators and immune characteristics between healthy and MM samples, as well as the possible influence of different m^5^C patterns, have important implications for understanding the immunoregulatory mechanisms of MM. Thus, in this study, m^5^C regulator expression analysis was systematically conducted in 60 healthy samples and 423 MM samples to examine correlations with immune characteristics. We identified m^5^C modification clusters and developed an m^5^C scoring system to predict and guide immunotherapy.

## 2 Materials and methods

### 2.1 Dataset collection and preparation

All gene expression profiles for samples were obtained from the Gene Expression Omnibus (GEO) database (https://www.ncbi.nlm.nih.gov/geo). Normative transformations and log2 transformations were applied to preprocess the raw data. A total of 483 samples (60 healthy samples and 423 MM samples) were obtained in the analysis, which included those from the GSE5900 (*N* = 22), GSE6477 (*N* = 81), GSE13591 (*N* = 138), GSE39754 (*N* = 176), GSE47552 (*N* = 46), and GSE80608 (*N* = 20). The array data of GSE5900 were obtained using the GPL570 platform (HG-U133_Plus_2 Affymetrix Human Genome U133 Plus 2.0 Array). GSE6477 and GSE13591 were obtained using the GPL96 platform (HG-U133A Affymetrix Human Genome U133A Array). GSE39754 was obtained using the GPL5175 platform (HuEx-1_0-st Affymetrix Human Exon 1.0 ST Array transcript (gene) version). GSE47552 was obtained using the GPL6244 platform (HuGene-1_0-st Affymetrix Human Gene 1.0 ST Array transcript [gene] version). Multiple probes were assigned to the same gene symbol, and median gene expression was calculated as the average expression level. “ComBat Batch correction” was applied to preprocess the value of the expression using the R package “sva” to remove batch effects (Leek et al., 2012).

### 2.2 m^5^C methylation regulator detection

The list of m^5^C regulators we used was derived from previously published data, and a total of 16 acknowledged m^5^C regulator genes were curated and analyzed to identify distinct m^5^C methylation modification patterns (Huang et al., 2021; Lv et al., 2021; Pan et al., 2021; Yu et al., 2021). The 16 m^5^C regulators included five writers (DNMT3A, DNMT3B, DNMT1, TRDMT1, and NSUN5), nine readers (MBD1, MBD2, MBD3, MBD4, MECP2, NTHL1, SMUG1, UNG, and YBX1), and two erasers (TET3 and TDG). The STRING (www.string-db.org) online database was used to build the protein–protein interaction (PPI) network among the m^5^C regulatory genes. Pearson’s correlation analysis was performed to examine the association among different m^5^C-related genes. Significant correlations were signified by a correlation coefficient *r*-value between −1 and +1. Visualization of the correlation matrix was performed using the “corrplot” package. We then performed expression analysis of m^5^C regulators between healthy and MM samples using the package “limma.” The boxplot and heatmap were generated using the “ggpubr” package and the “pheatmap” package, respectively.

### 2.3 Generation of the random forest algorithm for the m^5^C regulator and establishment of the nomogram

To establish an optimal training model that can predict the occurrence of MM, we constructed support vector machine (SVM) and random forest (RF) algorithm models using the “kernlab” and “DALEX” packages. An evaluation of the model was conducted using “reverse cumulative distribution of relative values,” alongside “boxplots of relative values”. We then used ten-fold cross-validation to validate the RF model. The RF algorithm was applied to rank the importance of m^5^C regulators using the “randomForest” package. A nomogram model was then built and developed by the “rmda” and “rms” packages to represent the selected m^5^C regulators. Furthermore, calibration curves were used to verify the consistency between actual and predicted occurrences of MM. We performed a decision curve analysis (DCA) to analyze the occurrence curve and evaluate whether the model offered beneficial decisions for MM patients (Iasonos et al., 2008). DCA is a method to evaluate clinical predictive models, diagnostic tests and molecular markers.

### 2.4 Correlation between m^5^C regulators and immune characteristics

Single-sample gene set enrichment analysis (ssGSEA) was performed to calculate an enrichment score, which estimates the relative abundance of each immune characteristic in each sample (Zhao et al., 2022). Immune characteristics consist of infiltrating immune cells (Zhang et al., 2020) and response reactions. The immune response reaction and related gene sets were accessed via ImmPort (http://www.immport.org). We derived enrichment correlations of differentially m^5^C regulators using the ssGSEA function to map the abundance of infiltrating immune cells and response reactions using the “GSEABase” package. Pearson’s correlation analysis was then used in order to calculate the correlation coefficient between m^5^C regulators and immune characteristics.

### 2.5 Consistent cluster analysis of m^5^C regulators

Based on the differential m^5^C regulators, unsupervised classification of the m^5^C dataset was performed for cluster analysis. Distinct m^5^C patterns were determined using “ConsensusClusterPlus” package in R, and the robustness of clustering was further systematically evaluated using a consensus clustering algorithm with 1,000 iterations (Wilkerson and Hayes, 2010). m^5^C regulator expression clusters were generated using principal component analysis (PCA). We subsequently evaluated the complex association between immune characteristics and different m^5^C clusters.

### 2.6 Pathway enrichment analysis of m^5^C clusters

Gene set variation analysis (GSVA) is a functional analysis to evaluate the enrichment of gene profiles through the expression dataset (Hänzelmann et al., 2013). The pathway gene sets, “h.all.v7.5.1 symbols” and “c2. cp.kegg.v7.5.1 symbols,” were obtained from the MSigDB database (http://www.gsea-msigdb.org/gsea/msigdb/index.jsp) and performed GSVA using the “GSVA” package to analyze pathway enrichment. Additionally, gene set enrichment analysis (GSEA) was applied to rank genes according to expression level, and we tested whether the gene set was enriched at the top or bottom of the ranking list (Subramanian et al., 2005). A normalized enrichment score (NES) was calculated as the primary statistic of GSEA. GSEA was conducted with the GSEA software (version 4.2.3) using the above official gene sets for enrichment.

### 2.7 Identification of m^5^C-related differentially expressed genes among three m^5^C clusters and biological function enrichment analysis

DEGs were screened among three distinct m^5^C clusters using the “limma” package, and m^5^C cluster-related genes were obtained by overlapping DEGs. Gene Ontology (GO) biological enrichment and Kyoto Encyclopedia of Genes and Genomes (KEGG) pathway enrichment were visualized using a bar plot and bubble plot. To further investigate m^5^C methylation modification in MM patients, an m^5^C score was calculated using the following formula (Zhang et al., 2020):

$${\text{m}}^{5}\text{C}\;\text{score}=\sum \left(PC1_{i}+PC2_{i}\right)$$ (1)

Where PC represents the principal component, and *i* represents the DEG expression.

### 2.8 Statistical analysis

Statistical analyses were performed using R x64–4.1.3. Quantitative data and normally distributed variables were compared using t-tests, and non-normally distributed variables were compared using the Wilcoxon rank-sum test. Comparisons of more than two groups of variables were performed using a one-way analysis of variance or the Kruskal–Wallis test. Pearson’s correlation analysis was used for the correlation analysis. A *p* < 0.05 was considered statistically significant. To control for false discovery rate (FDR), Benjamini–Hochberg was employed for multiple hypothesis testing. All statistical tests were two-sided.

## 3 Results

### 3.1 Landscape of m^5^C regulators in healthy and multiple myeloma samples

**FIGURE 1:**
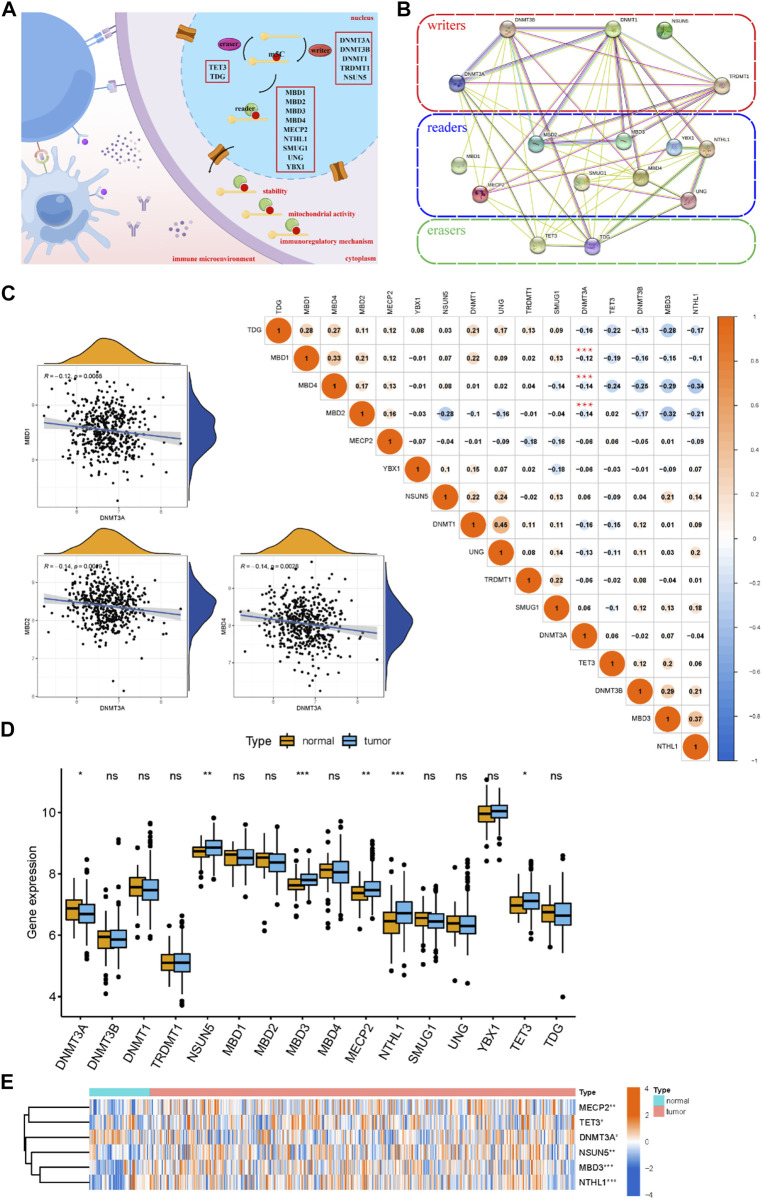
Landscape of m^5^C methylation regulators for healthy and MM samples. **(A)** The biological functions of m^5^C regulators, which are regulated by “writers,” “readers,” and “erasers” in the immune microenvironment. The diagram was drawn using Figdraw (https://www.figdraw.com). **(B)** Overview of the composition of m^5^C regulators and the PPI network among 16 m^5^C regulators in the STRING online database. **(C)** Correlation analysis of the m^5^C regulators. The size of each circle represents the correlation coefficient (negative values in blue and positive values in red). ***p < 0.001. Scatterplots show significant correlations between DNMT3A and MBD1, DNMT3A and MBD2, and DNMT3A and MBD4. **(D,E)** Boxplot and heatmap of the expression level of the m^5^C regulators for the healthy and MM samples.

We investigated 16 m^5^C methylation regulators, which included five writers (DNMT3A, DNMT3B, DNMT1, TRDMT1, and NSUN5), nine readers (MBD1, MBD2, MBD3, MBD4, MECP2, NTHL1, SMUG1, UNG, and YBX1), and two erasers (TET3 and TDG) (Figure 1A). The m^5^C regulators showed regular interactions via a PPI network (Figure 1B). The correlation analysis of the m^5^C regulators showed a close relationship between the m^5^C regulators, which suggested they function together (Figure 1C). The scatterplots between DNMT3A and MBD1 (*r* = −0.12), DNMT3A and MBD2 (*r* = −0.14), and DNMT3A and MBD4 (*r* = −0.14) showed significant negative correlations. Furthermore, the boxplot and the heatmap revealed that the expression of six significant m^5^C regulators in the MM samples compared to healthy samples (Figures 1D,E). Of all the 16 m^5^C regulators, we found that the expression of NSUN5, MBD3, MECP2, NTHL1, and TET3 was significantly upregulated, whereas DNMT3A was significantly downregulated in MM samples.

### 3.2 Construction of a nomogram to predict the occurrence of multiple myeloma

**FIGURE 2:**
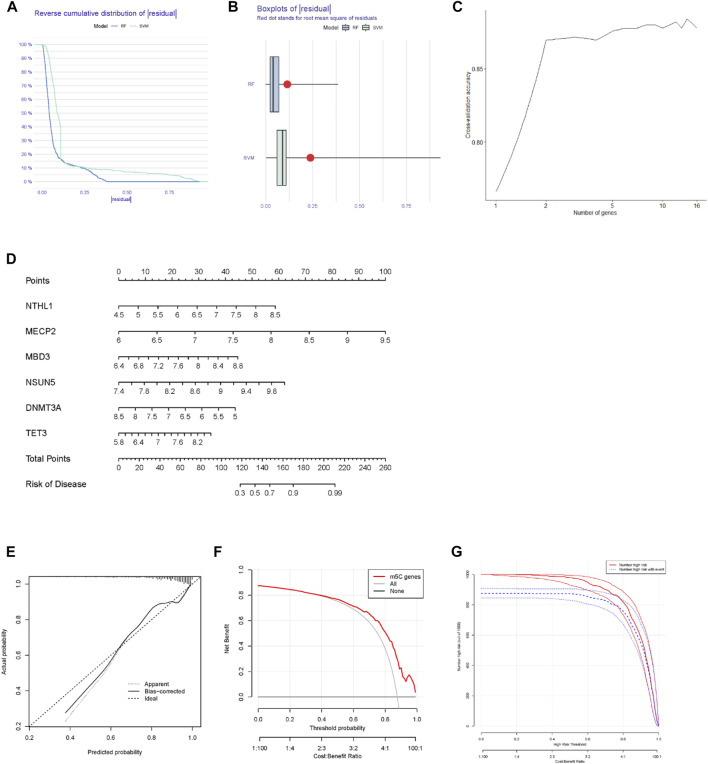
Construction of the nomogram to predict the occurrence of MM. **(A)** The RF model and SVM residual distributions were plotted using the reverse cumulative distribution of the residual. **(B)** Boxplots of the residual plot were obtained to test the residual distributions of the SVM and RF models. **(C)** Ten-fold cross-validation curve to assess the quality of MM prediction in the RF model. **(D)** Establishment of a nomogram model according to six differentially expressed m^5^C regulators. **(E)** The predictive ability of the nomogram model was evaluated and validated using a calibration curve. **(F)** Decision curves of the RF model of the m^5^C regulators. **(G)** Calibration curve of the nomogram for evaluating the occurrence of MM.

Based on 16 m^5^C regulators adopted in this study, we constructed SVM and RF models to predict the risk of developing MM. According to the “reverse cumulative distribution of the residual” (Figure 2A) and the “boxplots of the residual” (Figure 2B), the RF model provided the minimal residuals. Most samples in RF model have relatively small residuals, indicating that the RF model is better. Ten-fold cross-validation was applied to evaluate the accuracy of the RF model (Figure 2C). The curve indicated that when more than two genes were included in the RF model, the accuracy remained stable above 0.85. This led to the RF model being considered an accurate predictor of MM. Based on the differentially expressed m^5^C regulators, we then built and developed a nomogram to evaluate the risk of developing MM (Figure 2D). The nomogram indicated that higher expression of NTHL1, MECP2, MBD3, NSUN5, and TET3 would result in a higher probability of the occurrence of MM. Calibration curves were used to determine the accuracy of the nomogram model (Figure 2E). The DCA curve also suggested that the nomogram was beneficial to MM patients (Figure 2F). The abscissa of DCA curve is Threshold Probability. When the probability of a patient being diagnosed with MM reaches a certain threshold, it is defined as positive and treatment measures are taken. At this time, there will be benefits for treatment patient and losses for untreated patients. The net benefit on the ordinate is the result of the benefits minus the losses. For example, in Figure 2F, assuming that we choose a diagnosis of MM with a predicted probability of 0.8, then for every 100 patients predicted via “m^5^C genes”, about 50 would benefit from it. For every 100 patients predicted using “all genes”, 40 patients benefit from it. We can see that in the threshold probability range (0–1), the net benefit of m5C genes is higher than all genes. Furthermore, the clinical impact curve indicated that the nomogram model had remarkably high predictive power (Figure 2G).

### 3.3 Relationship between the m^5^C regulators and immune characteristics of multiple myeloma

**FIGURE 3:**
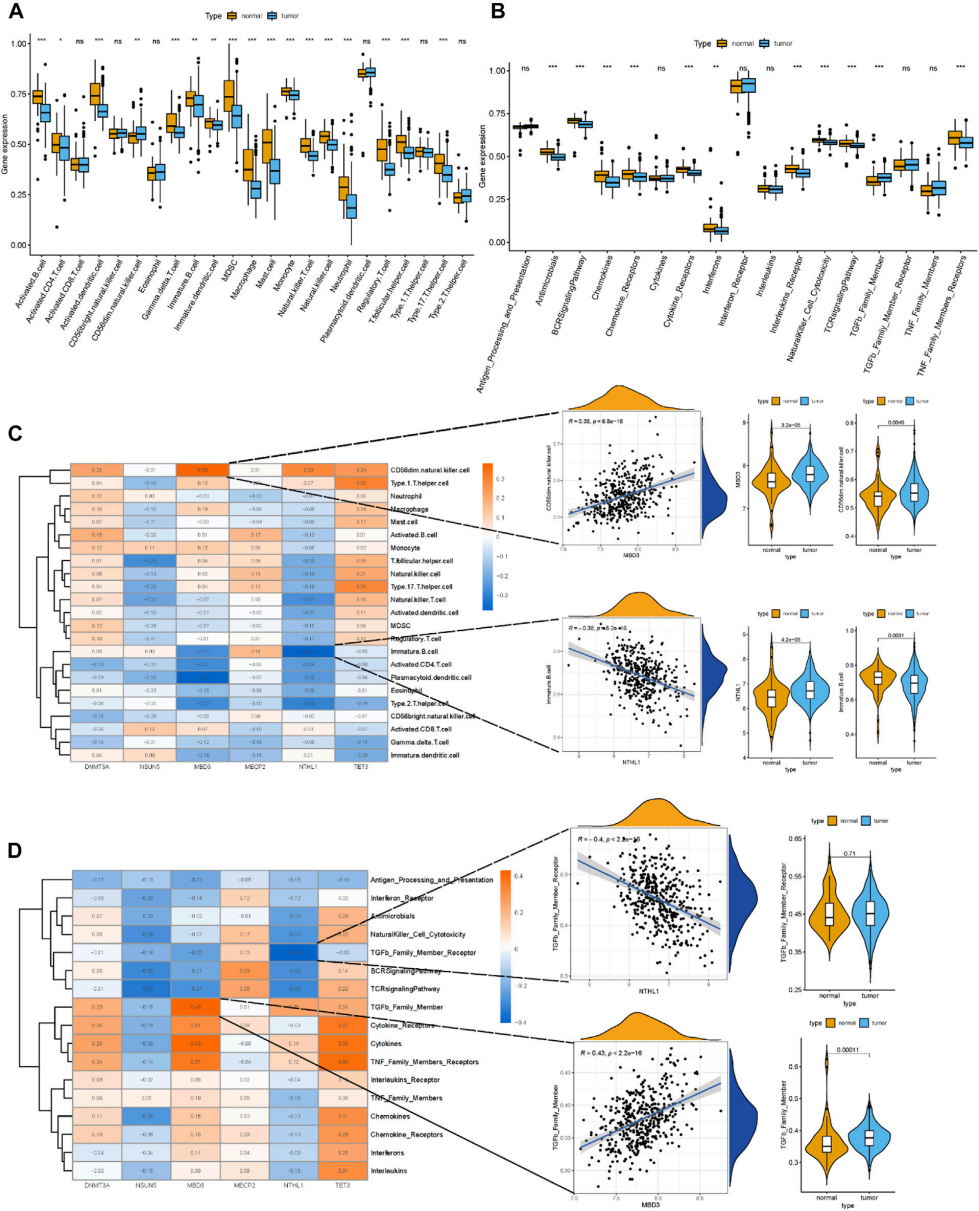
Relationship between m^5^C regulators and immune characteristics of MM. **(A)** Boxplot of the abundance of 22 immunocytes in the healthy and MM samples. **(B)** Boxplot of the abundance of 17 immune response reactions in the healthy and MM samples. **(C)** Heatmap showing correlations between the six m^5^C regulators and 22 immunocytes. The scatterplots showed the positive and negative correlations between m^5^C regulators and immunocytes. The expression data were presented in a violin box plot (right panel). **(D)** Heatmap showing correlations between the six m^5^C regulators and 17 immune response reactions. The scatterplots showed the positive and negative correlations between the m^5^C regulators and immune response reactions. The expression data were presented in a violin box plot (right panel).

Correlations between differentially expressed m^5^C regulators and immune cells and immune response reactions provide additional insight into the relationships between differentially expressed m^5^C regulators and immune characteristics. Based on immune expression profiles, numerous differentially expressed immune characteristics were identified between healthy and MM samples (Figures 3A,B). The correlation analysis revealed that several immune cells were closely associated with differentially expressed m^5^C regulators (Figure 3C). Among all the immunocytes, CD56 (dim) natural killer cells were most positively correlated to MBD3 (r = 0.38), and immature B cells were most negatively correlated to NTHL1 (r = −0.38), which indicated that the number of immune cells in MM patients is affected by differentially expressed m^5^C regulators. The expression level of CD56 (dim) natural killer cells and MBD3, immature B cells and NTHL1 also differed between healthy and MM samples. Additionally, the most immune response reactions were closely related to differentially expressed m^5^C regulators according to the correlation (Figure 3D). The transforming growth factor-β (TGFβ) family member was most positively correlated to MBD3 (r = 0.43), and the TGFβ family member receptor was most negatively correlated to NTHL1 (*r* = −0.4), which indicated that the number of immune response reactions is affected by differentially expressed m^5^C regulators.

### 3.4 Establishment of the m^5^C methylation modification clusters

**FIGURE 4:**
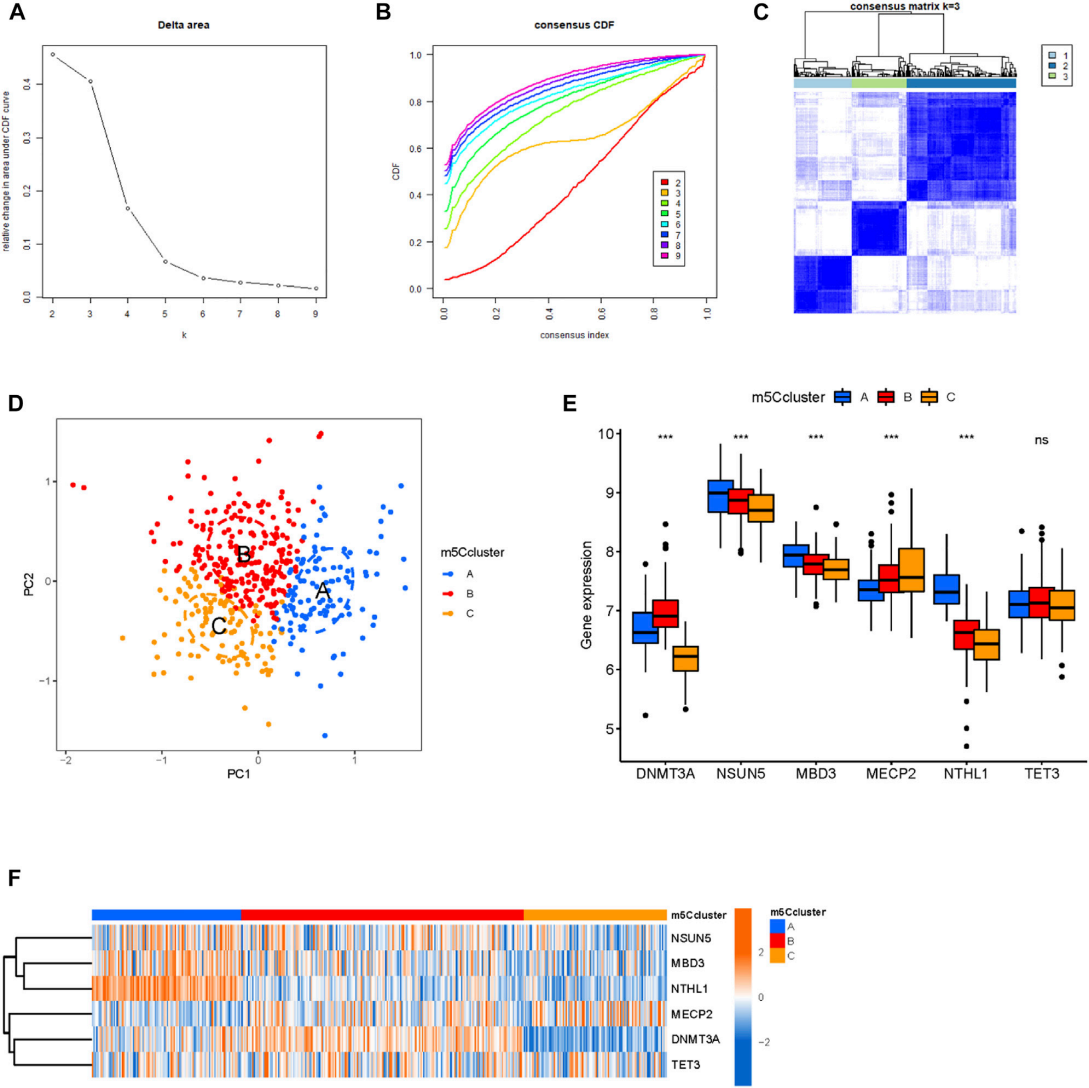
Establishment of m^5^C methylation modification patterns. **(A)** Consensus clustering of the cumulative distribution function (CDF) for k = 2–9. **(B)** Elbow plot showed the relative change in the area under the CDF curve. **(C)** The consensus heatmap defined three m^5^C clusters in 423 patients with MM. **(D)** PCA of the m^5^C clusters in MM patients. **(E,F)** Boxplot and heatmap showed the expression levels of m^5^C regulators among the three m^5^C clusters.

To establish the methylation modification clusters, a non-negative matrix factorization was applied based on the MM samples (Figures 4A–C). Three distinct clusters were classified as having significant populations in the PCA; namely, m^5^C cluster A, m^5^C cluster B, and m^5^C cluster C (Figure 4D). The boxplot and heatmap showed the high NSUN5, MBD3, and NTHL1 expression in m^5^C cluster A, high DNMT3A expression in m^5^C cluster B, and high MECP2 expression in m^5^C cluster C (Figures 4E,F).

### 3.5 Immune biological functional characteristics of the three distinct m^5^C clusters

**FIGURE 5:**
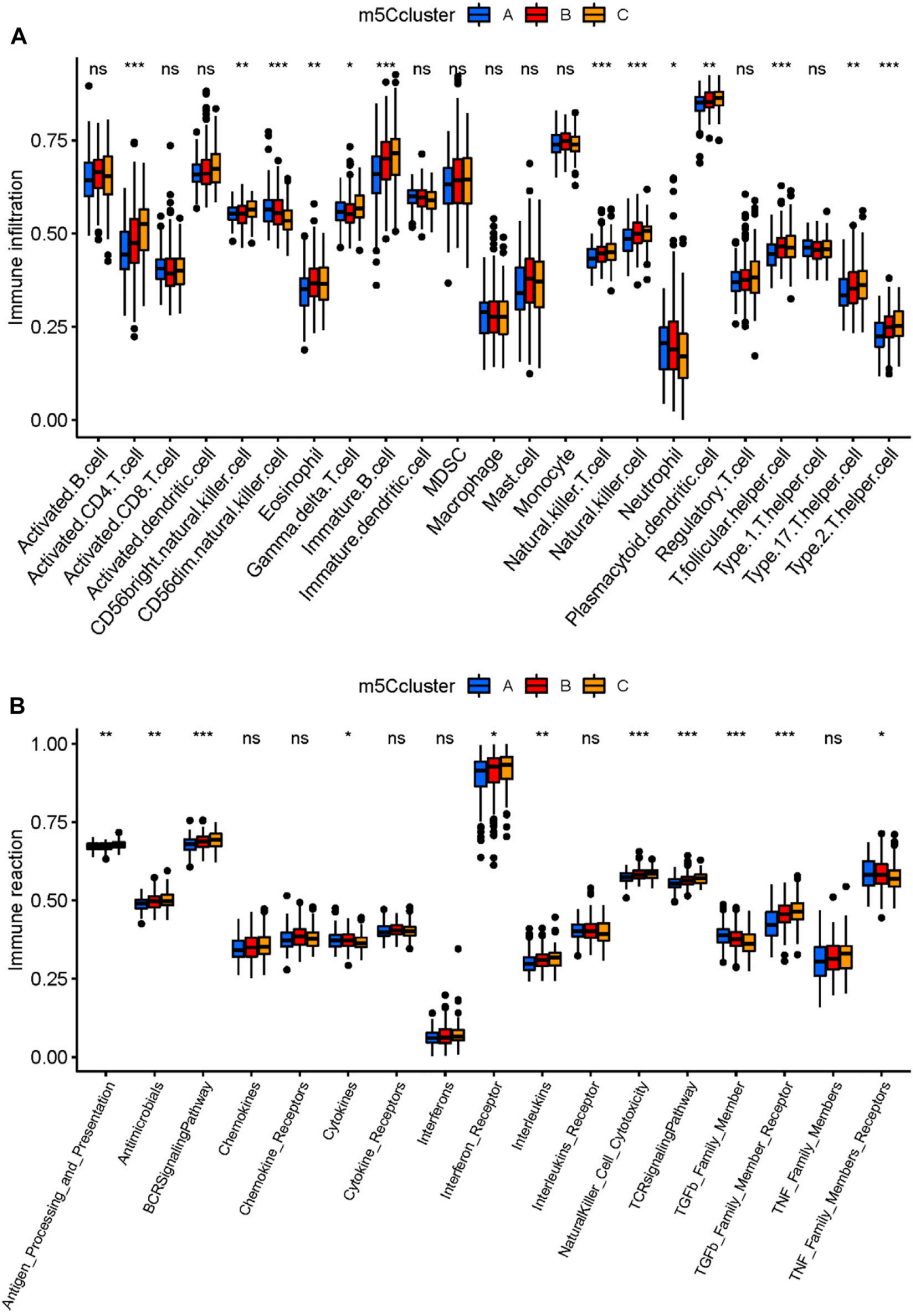
Immune characteristics of the three distinct m^5^C clusters. **(A)** Abundance of infiltrating immune cells in the three m^5^C modification clusters. **(B)** Differences in the 17 immune response reactions between the three m^5^C modification clusters. *p < 0.05; **p < 0.01; ***p < 0.001.

To deeply investigate the three m^5^C clusters, we compared the abundance of immune cells and immune response reactions. Results showed that various immunocytes differed in abundance in the three clusters (Figure 5A). Cluster A had a relatively higher number of immunocytes of CD56 (dim) natural killer cells and neutrophils than did clusters B and C. Cluster B had a higher level of eosinophil and T follicular helper cells, whereas active CD4^+^ T cells, CD56 (bright) natural killer cells, gamma delta T cells, immature B cells, natural killer T cells, natural killer cells, plasmacytoid dendritic cells, type17 T helper cells, and type 2 T helper cells were enriched in cluster C. As for immune response reactions, the TGFβ family member and tumor necrosis factor family member receptors were more active in cluster A than in clusters B and C. Antimicrobials and cytokines were more active in cluster B than in clusters A and C, and antigen processing and presentation, the B-cell receptor signaling pathway, interferon receptors, interleukins, natural killer cell cytotoxicity, the T-cell receptor signaling pathway, and TGFβ family member receptors were more active in cluster C than in clusters A and B (Figure 5B). These results suggested that cluster C played a more active role in immune characteristics. Taken together, our findings suggested that the m^5^C clusters possessed distinct immune characteristics and that differentially expressed m^5^C methylation regulators were critical for the regulation of the immune microenvironment in MM.

**FIGURE 6:**
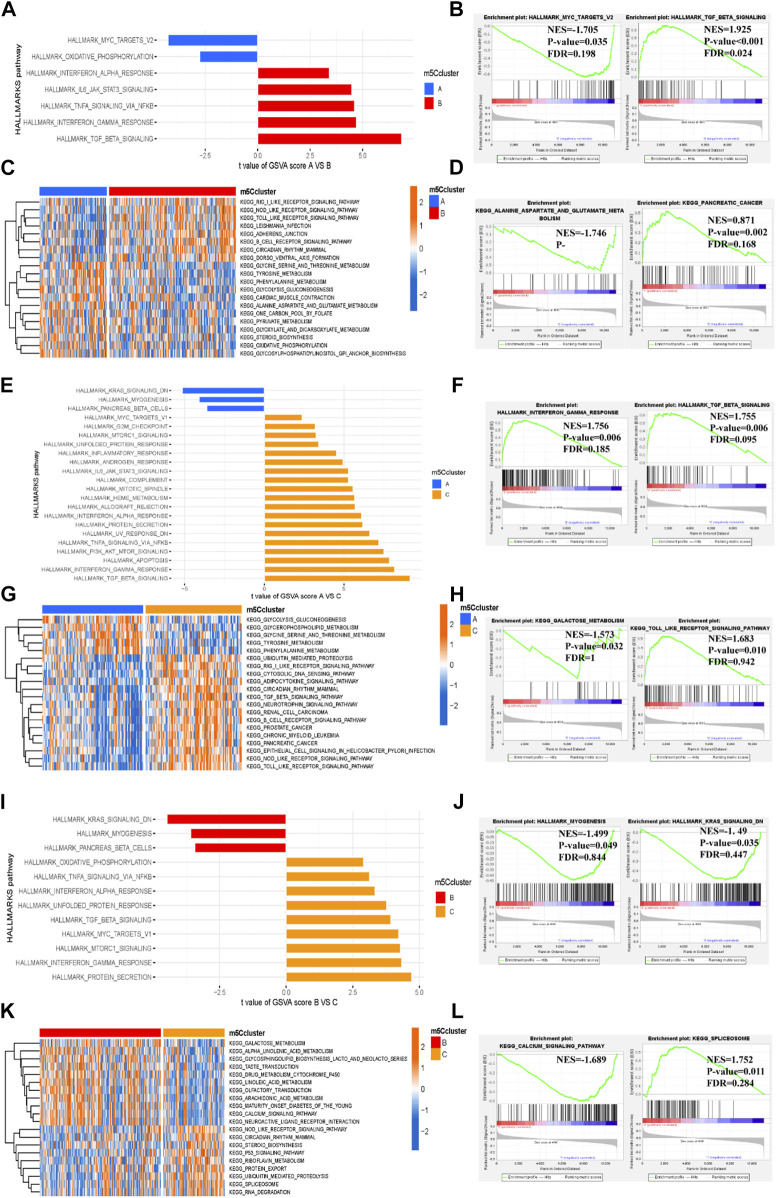
Biological functional characteristics of the three distinct m^5^C clusters. **(A–D)** HALLMARK pathway and KEGG pathway between m^5^C clusters A and B (**(A,B)** for HALLMARK pathway and **(C,D)** for KEGG pathway). **(E–H)** HALLMARK pathway and KEGG pathway between m^5^C modification clusters A and C (**(E,F)** for HALLMARKS pathway and **(G,H)** for KEGG pathway). **(I–L)** HALLMARK pathway and KEGG pathway between m^5^C modification clusters B and C (**(I,J)** for HALLMARKS pathway and **(K,L)** for KEGG pathway).

To further explore the biological functional characteristics of the three m^5^C clusters, we performed GSVA and GSEA to determine the enrichment of the biological pathways. The HALLMARK and KEGG pathways were compared between the clusters. Among the three m^5^C clusters, cluster C had more abundant HALLMARK and KEGG pathways, whereas cluster A had the fewest pathways (Figures 6A,C,E,G,I,K). Furthermore, the GSEA revealed that cluster A had the most significantly enriched terms related to the MYC targets V2 (NES = −1.705, *p* = 0.035, FDR = 0.198), and cluster B had the most enriched terms related to TGFβ signaling (NES = 1.925, *p* < 0.001, FDR = 0.024) in the HALLMARK pathway (Figure 6B). In the KEGG pathways, alanine aspartate and glutamate metabolism (NES = −1.746, *p* = 0.004, FDR = 0.447) were most enriched in cluster A, and pancreatic cancer (NES = 0.871, *p* = 0.002, FDR = 0.168) was most enriched in cluster B (Figure 6D). Similar enrichment pathways were observed in cluster C versus cluster A (Figures 6F,H) and cluster C versus cluster B (Figures 6J,L). After comprehensive GSVA and GSEA, we found that both methods enriched numerous similar HALLMARK and KEGG pathways.

### 3.6 Development of the m^5^C score and its biological functions

**FIGURE 7:**
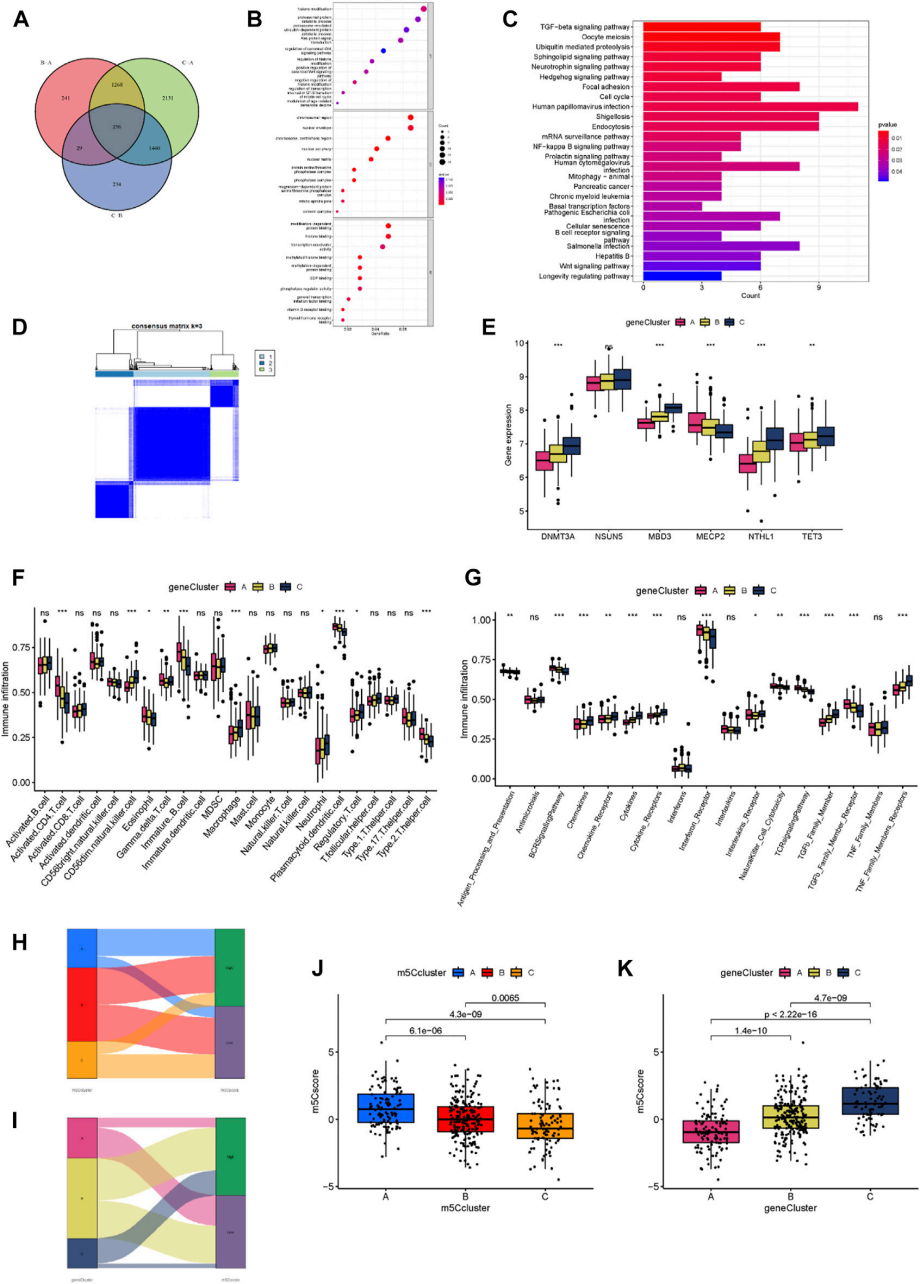
Development of the m^5^C score and its biological functions. **(A)** Venn diagram showing 256 m^5^C-related DEGs for the three clusters. **(B)** GO enrichment analysis of m^5^C-related DEGs. **(C)** KEGG enrichment analysis of m^5^C-related DEGs. **(D)** The consensus matrix heatmap defined three m^5^C gene clusters. **(E)** Expression levels of the m^5^C regulators for each of the three m^5^C gene clusters. **(F)** Abundance of infiltrating immune cells in each of the three gene clusters. **(G)** Differences in the activity of 17 immune response reactions among the three gene clusters. **(H)** A Sankey diagram of the different m^5^C modification clusters and m^5^C scores. **(I)** A Sankey diagram of the different m^5^C gene clusters and m^5^C scores. **(J)** Differences in the m^5^C scores among the three m^5^C modification clusters (*p* < 0.001). **(K)** Differences in the m^5^C scores among the three m^5^C gene clusters (*p* < 0.001). *p < 0.05; **p < 0.01; ***p < 0.001.

We performed the analysis of the DEGs of the three m^5^C clusters to confirm their potential biological functions and overlapped these DEGs to obtain the m^5^C cluster-related genes (Figure 7A). We identified 256 genes as m^5^C cluster-related genes, and we subsequently performed enrichment analysis. GO and KEGG enrichment results demonstrated that the tumor metabolism and immune-related pathways were enriched, which suggested that the m^5^C regulators exert a non-negligible role in MM (Figures 7B,C). To further examine the mechanism underlying the relationship between m^5^C and MM, we performed another consensus clustering analysis based on the 256 m^5^C cluster-related genes, and the samples were assigned to three distinct genomic patterns, which were named m^5^C gene clusters A, B, and C (Figure 7D). The boxplot showed statistically significant expression differences in the six significant m^5^C regulators among the three m^5^C gene clusters (Figure 7E). We then compared the abundance of immune cells and immune response reactions among the three m^5^C gene clusters. Results showed that most immunocytes and immune response reactions differed among the three clusters (Figures 7F,G). To predict the patterns of m^5^C gene clusters in the MM samples and assess the risk level of these patients, we implemented an m^5^C scoring scheme to assess m^5^C regulator expression patterns in MM samples and a Sankey plot to depict the score distribution (Figures 7H,I). Most samples of m^5^C cluster A are high risk, and most samples of gene cluster A are low risk; most samples of m^5^C cluster C are low risk, and most samples of gene cluster C are high risk. We then analyzed the differences in m^5^C scores among the m^5^C and gene clusters. Results revealed notable differences in m^5^C scores among the m^5^C clusters. The median of m^5^C cluster C was the lowest, and that of m^5^C cluster A was the highest, which indicated that a low m^5^C score was closely related to immune characteristics (Figure 7J). The m^5^C gene cluster C had the highest median m^5^C score, and gene cluster A had the lowest (Figure 7K).

## 4 Discussion

MM is a malignant tumor derived from terminally differentiated B lymphocytes and is characterized by the clonal proliferation of a large number of plasma cells in MM patients’ bone marrow (Das et al., 2022). MM tumor cells can remodel the tumor microenvironment and establish an immunosuppressive tumor microenvironment, which promotes the occurrence and development of MM (Minnie and Hill, 2020; Casey and Nakamura, 2021). The development of next-generation sequencing technology has demonstrated that m^5^C modification affects various disease processes, such as genomic stability, cancer cell differentiation, and inflammatory responses (Xue et al., 2020). Therefore, we believed that similar results of m^5^C modification could be observed in the MM immune microenvironment. To test this hypothesis, we used six GEO databases to obtain 60 healthy samples and 423 MM samples. Our study is the first study focused on m^5^C regulators in MM and shedding light on the links between m^5^C regulation and immune characteristics.

Firstly, we assessed the expressed gene profile of m^5^C regulators in the healthy and MM samples, and the differences indicated that m^5^C regulators were involved in MM progression. We also identified an RF model and selected six candidate m^5^C regulators (NTHL1, MECP2, MBD3, NSUN5, DNMT3A, and TET3) from the 16 m^5^C regulators to predict the occurrence of MM. A nomogram model to accurately predict the occurrence of MM was constructed, and the DCA curve demonstrated favorable calibration and benefit to MM patients when the nomogram was applied. DNMT3A plays a special role in the initiation of chromatin remodeling and gene expression regulation and is responsible for methylation pattern acquisition (Chen and Zhang, 2020). DNMT3A mutation carriers are characterized by increased expression of the T-cell alpha receptor constant chain and may be involved in monocyte-T-cell interactions (Abplanalp et al., 2021). NTHL1, MECP2, and MBD3 all have a binding domain that specifically maintain stability (Yano et al., 2006; Senarisoy et al., 2020). TET3 can catalyze the demethylation of m^5^C methyl groups under the synergistic effect of α-ketoglutarate and Fe^2+^ (Shen et al., 2021). It has not been reported whether any of the six differential m^5^C regulators are associated with MM. In m^5^C modifications, a methyl group attached to the fifth carbon of the cytosine ring in DNA and RNA molecules. This modification was first identified on DNA and later on RNA in the 1970s (Dubin and Stollar, 1975; Zin’kovskaia et al., 1978). For instance, MBDs could bind m^5^C and convert the methylation pattern information into appropriate functional cellular states. Chromosomal binding experiments indicate MBD-R2 and MECP2 associate on shared genomic loci (Gupta et al., 2016). All readers that “read” the information contained in these modifications to maintain stability and participate in translation and splicing.

We also investigated the correlation between m^5^C regulators and immune characteristics. For immunocytes, CD56 (dim) natural killer cells were most positively correlated with m^5^C regulators. The prognosis of MM patients has been shown to be influenced by CD56 (ElMenshawy et al., 2021), and DNMT3A-mutated AML patients have higher expression of CD56 (Hájková et al., 2012). The link between m^5^C regulation and the immune system suggests a close relationship between CD56 natural killer cells and MM. A set of immune reactions was correlated with m^5^C regulators, specifically the TGFβ family member and its receptor. The TGFβ family is a multifunctional cytokine and has long been recognized as an immune-suppressive factor (Tschernia and Gulley, 2022). It plays a major role in the beneficial immunosuppressive microenvironment of MM patients’ bone marrow niches (Alabanza et al., 2022), which indicates that m^5^C regulators are crucial to the progress of MM. However, there is no clear correlation between immune characteristics and m^5^C regulators. This may be due to the previous detection techniques limitations. For RNA sequencing, samples contain extremely few immune cells, so their abundance may not accurately reflect infiltration of immune cells (Stewart et al., 2020).

Our results further identified three m^5^C clusters with different immune characteristics and metabolism-related pathways. This immune pattern clustering approach can help us understand the underlying mechanism of immune regulation, allowing for the application of more precise molecular therapeutic approaches by clustering MM at the immune level, rather than simply at the phenotypic level. Various studies have confirmed that this strategy is feasible. For non-tumor diseases, several studies have applied this clustering method to study the impact of the immune microenvironment on disease (Zhang et al., 2021; Zhao et al., 2021). For tumors, one study divided gastric cancer into three patterns and demonstrated the impact of the tumor microenvironment on the disease, which offered new ideas for immunotherapy (Zhang et al., 2020). In our study, among the three different m^5^C clusters, cluster C had more immune characteristics and metabolism-related pathways than did clusters A and B. For example, cluster C was characterized by a higher number of immunocytes and immune response reactions, which included a greater abundance of plasmacytoid dendritic cells (pDCs) and interferon receptors, the former of which are increased in MM patients’ bone marrow and play a key role in the progression of MM (Ray et al., 2015). Moreover, pDCs are observed in m^5^C regulators, including the DNMT3A and NSUN family, and are regulated by interferon receptors (Bi et al., 2018; Fang et al., 2022). All three m^5^C clusters may have abundant metabolism-related pathways, which can help guide the identification of key m^5^C regulators and immune characteristics of MM. It may prove that MM can be classified as an alternate pathobiology-based classification based on the three distinct m^5^C clusters, which is related to clinical symptoms of the disease.

Finally, the m^5^C cluster-related genes and m^5^C gene clusters were identified. Given the need for individualized immunotherapy strategies for MM patients and improved understanding of m^5^C clustering patterns, developing a new m^5^C scoring system is urgently needed. We developed a scoring system to assess m^5^C regulator expression patterns in MM patients according to a previous study (Zhang et al., 2020). Based on the results of the m^5^C cluster-related genes, MM patients were divided into three m^5^C gene clusters. These genes’ expression may be influenced by m^5^C modification, and uncovering their biological functions helps to explain MM pathogenesis from the perspective of m^5^C modification. The m^5^C gene cluster C had the highest median m^5^C score. It has more activation in cytokines, and at the same time more CD56dim natural killer cells are seen in m^5^C gene cluster C. Most differential expressed m^5^C regulators are also up-regulated in m^5^C gene cluster C and MM. These results may suggest m^5^C regulators, cytokines, CDR56dim natural killer cells and MM have correlations. Our study reveals many similar correlations due to the abundance of results and a major scientific benefit of our study is helping other researchers identify key immune and m^5^C regulator features in MM quickly. Our study has this significance as one of its most important scientific implications.

However, there are some limitations in our study. More clinical data for each patient was not available, such as treatment and prognosis, for the longitudinal analysis. Correlation analysis of m^5^C clusters, pathological stages, and other clinical characteristics could not be performed on all samples. Besides, our findings were obtained mainly through bioinformatics analysis and require verification in subsequent single-cell sequencing and other experiments. Nevertheless, our findings provide new insights into the relationship between m^5^C regulators and the immune microenvironment of MM.

In conclusion, our study identified six differential m^5^C regulators and then built a nomogram to precisely predict the occurrence of MM. The three m^5^C clusters were shown to significantly impact the immune microenvironment and biological functional pathways, and these findings will be valuable for further progress in immunotherapy. Comprehensive analyses of m^5^C regulators in MM may offer a promising future for effective therapeutic strategies.

## Data Availability

The datasets presented in this study can be found in online repositories. The names of the repository/repositories and accession number(s) can be found in the article/Supplementary Material.
